# Accessibility of Virtual Primary Care for Adults With Intellectual and Developmental Disabilities During the COVID-19 Pandemic: Qualitative Study

**DOI:** 10.2196/38916

**Published:** 2022-08-22

**Authors:** Avra Selick, Janet Durbin, Yani Hamdani, Jennifer Rayner, Yona Lunsky

**Affiliations:** 1 Institute for Health Policy, Management and Evaluation University of Toronto Toronto, ON Canada; 2 Azrieli Adult Neurodevelopmental Centre Centre for Addiction and Mental Health Toronto, ON Canada; 3 Provincial System Support Program Centre for Addiction and Mental Health Toronto, ON Canada; 4 Department of Psychiatry University of Toronto Toronto, ON Canada; 5 Department of Occupational Science & Occupational Therapy University of Toronto Toronto, ON Canada; 6 Alliance for Healthier Communities Toronto, ON Canada; 7 Centre for Studies in Family Medicine Western University London, ON Canada

**Keywords:** COVID-19, intellectual disability, developmental disability, qualitative, telemedicine, virtual care, primary care

## Abstract

**Background:**

The COVID-19 pandemic has led to an unprecedented increase in the delivery of virtual primary care. Adults with intellectual and developmental disabilities (IDDs) have complex health care needs, and little is known about the value and appropriateness of virtual care for this patient population.

**Objective:**

The aim of this study was to explore the accessibility of virtual primary care for patients with IDDs during the pandemic.

**Methods:**

We conducted semistructured interviews with 38 participants in Ontario, Canada between March and November 2021. A maximum variation sampling strategy was used to achieve a diverse sample including 11 adults with IDDs, 13 family caregivers, 5 IDD support staff members, and 9 primary care physicians. An iterative mixed inductive and deductive thematic analysis approach was used to code the data and synthesize higher-level themes. The analysis was informed by the Levesque Patient-Centered Access to Health Care Framework.

**Results:**

We identified themes related to 4 of 5 access-to-care dimensions that highlighted both the benefits and challenges of virtual care for adults with IDDs. The benefits included saving time spent traveling and waiting; avoiding anxiety and challenging behavior for patients who struggle to attend in-person visits; allowing caregivers who live far away from their loved ones to participate; reducing illness transmission; and allowing health care providers to see patients in their home environments. The challenges included lack of access to necessary technology, lack of comfort or skill using technology, and lack of nonverbal communication; difficulty engaging and establishing rapport; patient exclusion from the health care encounter; and concerns about privacy and confidentiality. An overarching theme was that “one size does not fit all,” and the accessibility of virtual care was dependent on the interaction between the following 5 categories of factors: patient characteristics, patient context, caregiver characteristics, service context, and reason for a particular primary care visit. Though virtual care was not always appropriate, in some cases, it dramatically improved patients’ abilities to access necessary health care.

**Conclusions:**

This study suggests that a flexible patient-centered system including multiple delivery modalities is needed to ensure all patients have access to primary care. Implementing this system will require improved virtual care platforms, access to technology for patients and caregivers, training for primary care providers, and appropriately aligned primary care funding models.

## Introduction

In March 2020, the COVID-19 pandemic led to an unprecedented increase in the delivery of virtual primary care in countries around the world [1-6]. In Ontario, Canada, virtual care increased 56-fold to comprise over 70% of primary care in the first 4 months of the pandemic [2]. Though virtual care is sometimes defined broadly to refer to any use of technology to improve health care, this study focuses on technology-supported interactions between health care providers and patients in different locations. This includes synchronous and asynchronous interactions using video, telephone, and text-based technologies [7]. The rapid expansion of virtual care during the pandemic has raised questions about the quality and accessibility of virtual care for different patient groups [8,9]. Primary care is often the first point of access to the health care system and plays an important role in improving health outcomes, reducing health inequities, and reducing health care costs [10]. Considering the ongoing role of virtual care, it is critical to ensure that primary care remains accessible for all patients.

Adults with intellectual and developmental disabilities (IDDs) are a group that may require additional consideration to ensure that the increased use of virtual modalities does not compromise their access to care. The term IDDs is an umbrella term that includes a wide range of conditions of childhood onset that impact cognitive and adaptive functioning across the lifespan [11]. The conditions include, for example, intellectual disabilities, autism, Down syndrome, and fetal alcohol spectrum disorders. People with IDDs are more likely to live in poverty [12] and may therefore have greater challenges accessing technology. Some adults with IDDs live in congregate settings where there is limited access to technology and limited private space to use that technology, and where support staff may have limited skills to support technology use [13,14]. Other adults with IDDs may be supported by older parents with limited technology skills [15,16]. Additionally, some people with IDDs rely on facial expressions, lip reading, sign language, or communication devices for effective communication, which may be more difficult to use in virtual interactions [17,18]. Conversely, some people with IDDs find travelling to the health care appointment and waiting in the waiting room to be extremely stressful [19-22], and the ability to access care from the comfort of their own home may improve accessibility.

There is currently limited research on the accessibility of virtual primary care for adults with IDDs. A recent scoping review [23] on virtual health care for adults with IDDs identified 12 studies on access to virtual care, none of which focused on primary care. The review found that study participants generally reported high acceptability of virtual care, though the studies conducted during the pandemic reported more mixed feedback. The main challenges reported were related to participant skill and comfort using technology, and poor internet quality. The review concluded that the limited available literature suggests that virtual care can be accessible for adults with IDDs, but a better understanding is needed of when and for whom virtual care is appropriate. It is important to note that many of these studies were conducted prior to the pandemic. In these studies, patients typically opted for virtual care, care was usually provided by video, and access to technology was a requirement for participation. During the pandemic, virtual care was sometimes the only option, and it was much more likely to be delivered by telephone than video [24,25]. Additionally, in some studies, patients received virtual care in supported settings (eg, a telemedicine clinic) versus the typical experience during the pandemic where patients participated in virtual care from their homes.

We identified one study focused on video-based virtual primary care for autistic adults during the pandemic [26]. The study identified benefits to virtual care, including increased patient comfort and reduced risk of COVID exposure, and challenges, including technology issues, lack of a physical examination, and reduced patient engagement (eg, distracted and wandered away from the visit). Participants in this study reported that they found communication via virtual care to be the same or better than in-person communication, though the sample did not include individuals with intellectual disabilities. In contrast, other studies on virtual interactions for people with IDDs during COVID-19 found that effective virtual communication could be challenging [27,28].

The aim of this study was to explore the accessibility of virtual primary care for patients with IDDs. Our intention was to understand the experiences of virtual primary care in Ontario, Canada during the pandemic to help inform the potential ongoing role of virtual care within an accessible primary care system.

## Methods

### Access to Care Framework

We conceptualized access to care based on the Patient-Centered Access to Care Framework developed by Levesque et al [29]. In this framework, access is defined as the fit between the characteristics of the service and the needs and abilities of the individual. The Access to Care Framework identifies 5 dimensions of service accessibility with 5 corresponding dimensions reflecting the patient’s ability to access the service as follows: (1) *approachability*, how easy the service is to identify or be aware of, and a*bility to perceive*, the patient’s awareness of their need for services; (2) *acceptability*, whether the service is perceived to meet patient needs, and *ability to seek*, the patient’s capacity to seek care; (3) *availability and accommodation*, how, when, and where services are offered, and *ability to reach*, the patient’s ability to use the service; (4) *affordability*, the cost of the service, and a*bility to pay*, the patient’s financial resources; and (5) *appropriateness*, the quality of the service, and *ability to engage,* the patient’s motivation to engage in care.

### Methodology

Qualitative description methodology was used to guide the overall study design. Qualitative description focuses on describing and understanding participant experiences with the goal of achieving descriptive and interpretive validity [30-32]. This pragmatic participant-centered approach is recommended for applied health services research aimed at informing policy and practice [33] and for studies focused on addressing health disparities for vulnerable populations [34].

### Sampling and Recruitment

This study included adults with IDDs, caregivers (including family members and IDD support staff members), and primary care providers. The study was restricted to participants living in Ontario, Canada who had experience participating in at least one virtual primary care visit for an adult with an IDD over 18 years of age and had the capacity to provide informed consent. Adults with IDDs were included if they self-identified as having an IDD. Given the limited prior research on this topic, we used a maximum variation sampling strategy to achieve a diverse study sample with the intention of capturing a wide range of experiences [35,36].

Efforts were made to recruit a demographically diverse set of participants based on age, gender, and geographic location across the province. Efforts were also made to recruit primary care providers from different practice models, as they may have different resources available to support virtual care, and adults with IDDs living independently, with family, and in supported settings. To achieve these aims, we used broad recruitment strategies. A study flyer was developed and shared widely using existing health care provider networks (eg, the Alliance for Healthier Communities, the Association of Family Health Teams of Ontario, and Developmental Disabilities Primary Care Program), community agencies (eg, Vita Community Living Services, Community Living Ontario, and Surrey Place Centre), caregiver and self-advocate networks (eg, the Azrieli Adult Neurodevelopmental Centre self-advocate and caregiver advisories), social media, and other relevant newsletters (eg, Health Care Access Research and Developmental Disabilities newsletter and Developmental Services Ontario newsletter). All participants and recruitment contacts were encouraged to share the flyer widely. Additional targeted recruitment was conducted as needed to improve representation across participant groups.

### Data Collection

Semistructured interviews were conducted with participants by phone or the Webex video conference platform (Cisco) according to participant preference. Participants also had the option of typing their responses through the chat function on the Webex platform. All interviews were conducted by the first author (AS) who has 10 years of qualitative research experience, including prior experience conducting interviews with adults with IDDs.

Tailored interview guides were developed for each participant group informed by a previous scoping review of the literature [23] and the Levesque Access to Care Framework [29]. Questions focused on the experience of receiving, supporting, or delivering virtual care; preferences related to the future role of virtual care; and supports needed for successful virtual care. Virtual care was defined as including any care provided remotely including by phone, video, or written communication (eg, email). Demographic information on age, gender, disability, and geographic location was also collected. Interview questions for adults with IDDs were developed recognizing the unique considerations in interviewing this population, including the need to adapt language based on individual capacity, potential difficulty with abstract concepts and recalling past events, and risk of suggestibility or acquiescence [37-39]. If helpful, interview questions were sent in advance. Patients with IDDs had the option of being interviewed independently or with a support person. If both the patient with an IDD and their caregiver choose to participate in the study, dyadic interview techniques were used to elicit both the patient perspective, using caregiver-mediated communication if appropriate, and the caregiver’s own perspective [40].

Interviews lasted approximately 20-60 minutes and were audio recorded and transcribed. Field notes were taken during and immediately following each interview to document interviewer impressions and nuances that may not be captured in the recording [41]. An honorarium was provided to all participants.

Interviews were conducted between March and November 2021. In Ontario, temporary billing codes were implemented in March 2020 to reimburse physicians for virtual health care, and physicians were encouraged to take a “virtual first” approach [42,43]. The proportion of care delivered virtually in Ontario has fluctuated throughout the pandemic in accordance with each wave of COVID, but early data suggest that it consistently accounted for a substantial proportion of patient visits throughout the study period [43,44]. A recent Ontario study using population-level administrative data found that about 62% of adults with IDDs in Ontario used virtual care during the first year of the pandemic, similar to the proportion of adults without IDDs [45].

### Ethics Approval

This study received approval from the research ethics board at the Centre for Addiction and Mental Health (REB # 160/2020) and the University of Toronto (Protocol # 40483). All participants provided informed consent prior to participating in the study.

### Analysis

A mixed inductive and deductive thematic analysis approach was used to guide the analysis [46-48]. Though the study was informed by a pre-existing Access to Care Framework [29], the framework was being applied in a novel context, and it was important that it should not restrict or limit initial coding. Therefore, initial coding was guided by the research question, but remained relatively open and data driven. The first author (AS) developed an initial codebook based on a review of all transcripts and field notes. A subset of transcripts from each stakeholder group was reviewed and discussed with 2 additional authors (JD and YL) to identify key ideas and patterns of ideas, and to refine the initial codebook. The first author (AS) then coded all transcripts, iteratively updating and refining the codebook throughout the process. Coding was conducted using NVivo 12 software (QSR International).

A multi-stage process was used to synthesize the collated data and generate themes. First, an open data mapping exercise was conducted to explore relationships and patterns across all codes. Codes were then mapped onto the Access to Care Framework, considering fit with existing framework domains. These initial maps were reviewed and discussed by all the study authors, and key themes were identified. These initial themes were then reviewed, discussed, and refined with members of each stakeholder group (ie, self-advocates, caregivers, and primary care physicians) as part of a peer debriefing process [49]. Results are reported by access to care domain. Quotations are included to illustrate the findings.

### Rigor

This study was conducted as part of the doctoral thesis of the first author and was supported by a team that included researchers working in both the health care and IDD sectors, including health services researchers, a psychologist, an occupational therapist, and family members of people with IDDs. Our team engaged in ongoing critically reflexive dialogues to reflect on our positionalities and assumptions in relation to this work and to consider how they shaped the study findings [49,50]. The collective experiences and perspectives of this team guided and informed the study design, analysis, and interpretation.

Several strategies were used to support trustworthiness in this study [32,49]. Credibility was supported by promoting an open and safe interview process, using clear and easy-to-understand interview questions, and conducting a peer debriefing process to support data interpretation. Dependability was supported through the use of an audit trail to clearly document each step of the analysis process, and through detailed and transparent reporting of the findings. Transferability was supported by providing detailed descriptions of the sample and the recruitment process, and contextualizing findings through thick descriptions so readers can gauge the applicability to different settings. Confirmability was supported through the use of field notes to contextualize the data, an iterative coding process based on multiple re-readings of the data, and inclusion of quotations to illustrate the findings.

## Results

### Participants

In total, 38 individuals participated in this study, including 11 adults with IDDs, 13 family members, 5 IDD support staff members, and 9 primary care physicians. Participants included 25 women and 13 men (between 23 and 69 years old) living across the province (Greater Toronto Area, 19; Eastern Ontario, 9; Western Ontario, 8; Northern Ontario, 2). The study included adults with IDDs or the caregivers of adults with IDDs, who live with their family (n=18), independently (n=5), or in supported settings (n=6). Primary care physicians participated from all 4 primary care delivery models in Ontario: family health teams (n=3), community health centers (n=2), physician group practices (n=3), and solo practitioners (n=1). Seven of the nine physicians reported having practices with a particular focus on patients with IDDs. Participants had or supported people with a range of IDDs including autism, intellectual disabilities, and Down syndrome. Many of these individuals also had co-occurring health issues, including mental illness, vision and hearing impairments, physical disabilities, and chronic illnesses.

### Main Findings

**Figure 1:**
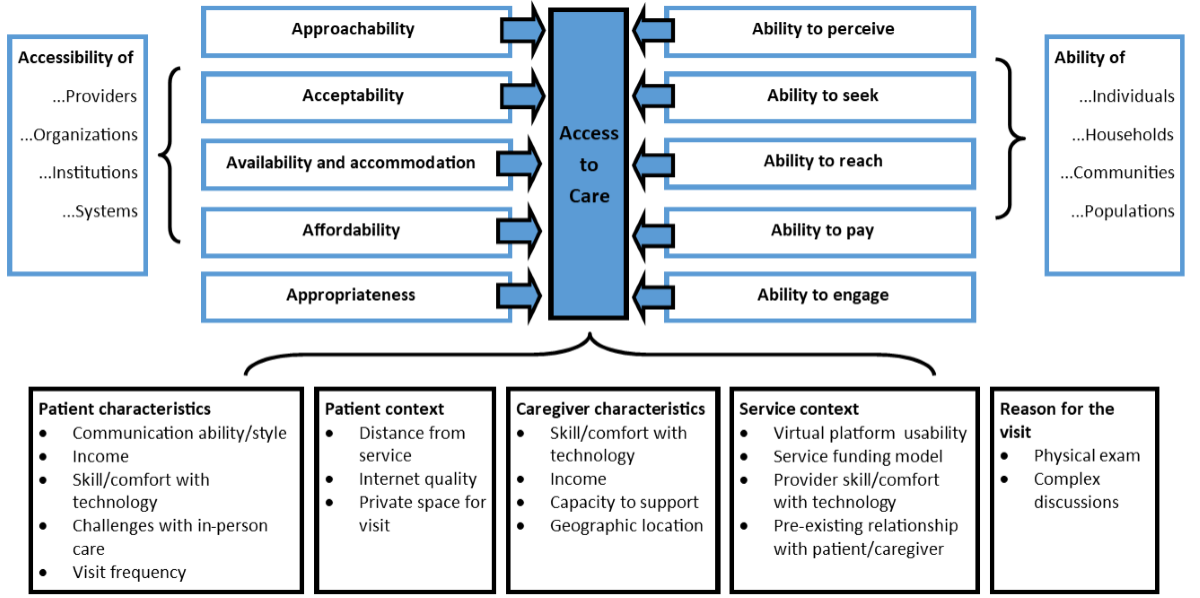
Access to virtual care for adults with intellectual and developmental disabilities.

All study participants reported receiving or delivering synchronous virtual primary care by telephone or video. These synchronous appointments were sometimes supported by asynchronous communication by email or text message to schedule appointments; ask questions; and send documentation, photographs, and videos. Across the 38 interviews, we identified themes that aligned with each of the access-to-care dimensions, with the exception of approachability/ability to perceive (see Table 1). This is likely due to the data source used for this study. Interview participants could only speak about services they had used (ie, you do not know what you do not know). These themes were informed by mutable and immutable variables related to the patient’s characteristics, the patient’s context, the caregiver’s characteristics, the service context, and the reason for a particular primary care visit (see Figure 1). Each of these themes is described in more detail below.

**Table 1:** Access to care dimensions and themes.

Dimensions and themes		Description
**Acceptability/ability to seek (patient/caregiver comfort or satisfaction with the services)**		
	Convenience	Virtual care saved time and could be more convenient than in-person care. Phone was seen as quick and easy; video could be more difficult and time-consuming.
	Change is hard	Virtual care was new, and change can be challenging.
	Health care visits as a valuable outing	For some patients, in-person visits were enjoyable outings and important opportunities to practice social skills that were lost with virtual care. Some patients had important rituals or reward systems to facilitate health care visits that were disrupted by virtual care.
	Caregiver distress	Virtual care sometimes put additional responsibility on the caregiver to negotiate health care interactions.
**Availability and accommodation/ability to reach (patient/caregiver ability to use the service)**		
	Technology quality, access, and skill	Patients and caregivers did not always have access to necessary technology. Patients, caregivers, and primary care providers sometimes lacked skill and comfort using technology. Switching between multiple virtual platforms was confusing for some patients and caregivers.
	Difficulty travelling and waiting	Virtual care facilitated access to care for patients unable or challenged to attend in-person visits.
	Participation in the visit	Virtual care facilitated participation of multiple care providers and caregivers in the visit. Patients were less likely to be included in virtual visits, especially by phone.
	Patient independence	Virtual care supported independence for patients unable to travel by themselves. However, it reduced independence for patients who could travel but required support to use technology.
**Affordability/ability to pay (affordability of the service for patients/caregivers)**		
	Travel and parking costs	Virtual care saved costs related to travel and parking.
	Staff time	Supported settings saved costs due to fewer staff required to accompany patients to their health care visits.
	Technology costs	Costs were incurred to purchase high-speed internet and internet-enabled devices. Residential settings incurred costs for technical support staff.
**Appropriateness/ability to engage (quality of care received by the patient)**		
	Communication and rapport	Nonverbal communication was lost in phone interactions and was more challenging in video interactions for patients, caregivers, and primary care providers. Communicating via phone could be more challenging for people with hearing impairments. It was sometimes difficult to manage conversations with multiple participants. The ability to use chat functions supported improved communication for some patients. Video was sometimes a better option to see facial expressions or read lips while masks were required for in-person visits. Some participants found it difficult to develop rapport virtually, especially with new primary care providers.
	Seeing patients at home	Participating in visits from their home made some patients more comfortable and less anxious, leading to more effective visits. Video visits allowed primary care providers to see patients in their home environment.
	Importance of physical examination	Physical examinations are sometimes a necessary component of care and were particularly important for some people with IDDs^a^ who could not describe their symptoms.
	Privacy/confidentiality	Patients did not always have a private space from which to participate in the visit. Some patients were concerned about cyber security. Some caregivers were concerned that people with IDDs may be more vulnerable to online scams and may disclose medical information inappropriately.
	Safety	Virtual care reduced the transmission of COVID-19 and other illnesses. Some patients felt safer in-person when discussing potentially triggering topics.

^a^IDD: intellectual and developmental disability.

#### Acceptability/Ability to Seek

Acceptability of virtual care varied widely across participants. There were those who preferred their health care appointments to take place by phone, by video, or in-person and some who had no strong preferences. The following 4 themes related to acceptability/ability to perceive were identified: convenience, change is hard, health care visits as a valuable outing, and caregiver distress.

##### Convenience

Some patients and caregivers appreciated the convenience of participating in care from home. Virtual visits allowed them to avoid sometimes lengthy travel time to appointments, potentially requiring missed work or school. Instead of waiting in waiting rooms, they could go about their normal day until the primary care provider called. A 29-year-old autistic woman shared:

It's so convenient…I think it's great that they can kind of assess you on the phone and then say, “OK, well, I think you need to come in or you don't need to come in.” […] It saves so much time for everyone- getting ready to go, finding my stuff, getting it together, taking the subway, waiting in the waiting room.

This was particularly important in cases where the patient or caregiver lived far away from the provider, or if the caregiver had other responsibilities (eg, multiple care recipients). Phone visits were seen as particularly fast and easy, while video calls could be more complicated and time-consuming for patients, caregivers, and primary care providers.

##### Change is Hard

Some patients, caregivers, and primary care providers disliked virtual care because it was new and different. Participants also reported that change is always challenging, but can be particularly challenging for some people with IDDs. A 48-year-old autistic man explained:

[Virtual care] is not something that I think should be encouraged for people on the spectrum because it's, yeah it's another change.

Participants noted that it is possible that some of the resistance or dislike of virtual care will change as people grow more accustomed to it.

##### Health Care Visits as a Valuable Outing

Some participants highlighted the experiences they or the person they support missed out on due to virtual care. Some people with IDDs enjoyed travelling for their health care visits and felt that virtual care deprived them of an enjoyable outing. For some people with IDDs, health care visits were an important opportunity to socialize and practice skills. A staff member at a group home in eastern Ontario explained:

Another big downfall [of virtual care] is just that so much of what we do here for these individuals is supporting that socialization and those social skills and it's one less opportunity for us to teach that in person. […] So for these individuals, speaking with the receptionist and having that opportunity to practice with the nurse and then the doctor, it was a lot of opportunities for them to practice that social interaction.

There was also concern that some people with IDDs will find it very challenging to return to in-person visits, and it may require significant work to rebuild tolerance and comfort with health care settings.

Some people with IDDs had important rituals or reward systems surrounding the health care visit that served as motivation and positive reinforcement to support the health care visit, which were disrupted by the shift to virtual care. The mother of a 26-year-old autistic woman with multiple chronic health issues shared:

[My daughter] geared herself to these appointments by, you know, deciding what she's going to wear and how she's going to get there. She's going to have a Starbucks latte afterwards or she's going to going into a special place after the appointment [...]. She makes it a special outing.

Without these rituals, some people with IDDs were less motivated or willing to participate in the visit.

##### Caregiver Distress

Some caregivers felt that virtual care placed additional pressure on the caregiver to make medical decisions, conduct assessments, and communicate on behalf of the person they support. While the provider could conduct a physical examination during an in-person visit, virtual care required the caregiver to relay the relevant information to the provider to the best of their ability, and in some cases, they needed to conduct elements of the examination themselves (eg, monitoring blood pressure). This caused some caregivers a great deal of additional stress. The mother of a 28-year-old man with Down syndrome explained:

I have enough on my plate trying to parent him, trying to help him […] manage every day, especially during COVID. To ask me to be the person that has to communicate all that to the medical person at the other end […] can get to be extremely overwhelming. [...] For a family that has a child or an adult with intellectual disabilities who may or may not be able to communicate their needs, you're asking those parents to make even more decisions or communicate more things. And what if I mess up? What if I miss something?

#### Availability and Accommodation/Ability to Reach

This domain includes themes focused on the benefits or challenges of virtual care in facilitating the patient’s and caregiver’s abilities to attend a primary care visit. The following 4 themes were identified: technology quality, access, and skill; difficulty travelling and waiting; participation in the visit; and patient independence.

##### Technology Quality, Access, and Skill

A number of technology-related challenges were raised by participants as barriers to video-based care. Internet or technology failure (eg, internet cuts out or camera stops working) interrupted appointments. This was particularly challenging in more rural areas where internet quality is poor. Some patients, caregivers, and primary care providers also lacked skills or comfort using technology. This was further complicated by the fact that currently there are a number of approved secure video platforms used for health care in Ontario, which was confusing for some participants. The concern was also raised that not all patients and caregivers have access to internet-enabled devices or high-speed internet necessary to conduct video-based appointments. For these reasons, phone was sometimes preferred to video, which could appear to be too challenging and time-consuming to use. One physician shared:

I don't think the video worked very well at all. It was a challenge to set it up, you had to give a lot of instructions on how to connect and turn it on. […] And I found that even if I have a [video] visit, I still have to call them on the phone to see what was going on. ‘Where are you?’ ‘Is it working?’ So it's just frustrating. And then there were also technical glitches, screen freezes, no sound. […] And also the quality, some people just don't have the Wi-Fi or Internet to have a great video quality so it’s just a blurry picture. It's not helpful anyway.

Participants also shared, however, that they have become more skilled and comfortable using technology during the pandemic. In particular, it was noted that the pandemic has demonstrated that people with IDDs are in many cases far more capable of using technology than was previously assumed. The mother of a 34-year-old woman with Down syndrome shared:

She is totally engaged [in virtual programs] and she's never done that before. She's never had the opportunity to try it this way. So this is a gift of COVID. All this came about because of COVID and people are seeing the benefits.

##### Difficulty Travelling and Waiting

There are some people with IDDs for whom getting to an in-person visit or waiting in the waiting room is prohibitively difficult due to physical, mental, or behavioral challenges. Participants highlighted that for these individuals, virtual care is not only convenient, but also critical to enable them to access care. One physician shared:

It's not just that their needs are convenience, their needs are accessibility needs. It is very difficult to get a patient that requires, you know, an hour and a half of transitioning and then can't manage the sensory overload experience of the waiting room and, you know, other complex issues and is brought to the appointment by someone who isn't even their main caregiver and doesn't know them. Those aren't convenience issues, those are accessibility, accommodation needs.

##### Participation in the Visit

Participants noted that virtual care also impacted the extent to which patients, caregivers, and other health care providers can participate in the primary care visit. Virtual care allowed multiple caregivers, especially family members who live far away from the person they support, to all attend the visit, thus improving lines of communication and supporting appropriate decision-making. The brother of a 55-year-old man with Down syndrome described the benefits of everyone being in the same virtual room as follows:

Everything is transparent and there's no miscommunications. The group home staff are hearing the same thing that we are hearing.

Virtual care also supported case conferencing with multiple health care providers, improving care quality and coordination.

Patients, however, were less likely to be included in virtual primary care visits, particularly if they took place by phone. Sometimes it did not occur to the caregiver or provider that the patient could or should be included. The mother of a 24-year-old autistic man explained:

Because it was a phone consult, I don't even think we thought of it to be honest.

Sometimes the patient was uninterested or harder to engage in the interaction. The sister of a 38-year-old autistic man shared:

He's not a part of it, no. He's nonverbal and he doesn't really communicate. We tried video […] and he would sit down for maybe two minutes and then he just wants to bolt away. It doesn't keep his attention.

On the other hand, it was noted that a benefit of virtual care is that patients have the flexibility to come and go as they wish. A physician explained:

Even with the video calls, it's very rare that the patient will stay on the call the entire time. But I think that's OK, too. In some ways it's nice if I can see them and then the parents can give me the history and tell me more about what's been going on.

It was also suggested that it may be appropriate in some cases for the patient not to be included in the virtual visit. Examples were shared of successful hybrid approaches where an initial phone call with the caregiver was used to gather information, and then, a shorter in-person appointment was conducted with the patient, which was easier for them to tolerate.

##### Patient Independence

Participants appreciated that virtual care can support independence for some people with IDDs who may need help with transportation but could participate in a virtual visit independently. A 30-year-old autistic man shared:

I don't drive, [my mom] drives. So it's basically on her to get me to the [clinic]. So it's better for me and her when I have an appointment and I don't have to physically go there.

Conversely, there were some people with IDDs who could attend in-person appointments by themselves but required support for virtual encounters.

#### Affordability/Ability to Pay

This domain includes the following 3 themes related to the additional costs or costs saved due to virtual care: travel and parking costs, staff time, and technology costs. This domain received less focus in the interviews, potentially due to the challenges of isolating costs of virtual health care from the general increase in virtual interaction during the pandemic.

##### Travel and Parking Costs

Participants appreciated costs saved with virtual care due to avoided travel and parking costs. This is especially relevant for people who live further away from their primary care provider or in large urban centers with high parking costs.

##### Staff Time

Participants suggested that group homes or other supported settings may save costs due to fewer staff members needed to support in-person appointments.

##### Technology Costs

Participants reported that video appointments required increased spending on high-speed internet and internet-enabled devices and on information technology support in group home settings. However, it was noted that the increased spending on technology was not solely due to virtual health care. Some patients also incurred costs for medical equipment needed to support at-home monitoring (eg, blood pressure monitor, pulse oximeter, and scale).

#### Appropriateness/Ability to Engage

This domain includes findings related to service quality and effectiveness. The following 5 themes related to this domain were identified: communication and rapport, seeing patients at home, importance of the physical examination, privacy and confidentiality, and safety.

##### Communication and Rapport

In some cases, virtual care can be a barrier to effective communication. Some people with IDDs were more reliant on nonverbal cues or body language for communication, which was lost entirely in phone interactions and was still sometimes challenging over video. The father of a 22-year-old autistic man explained:

Because of the autism, he doesn't pick up on the behavior cues as well virtually. […] There's definitely a disconnect between what's... what he's able to process, and I don't think he gets as good cues. You need a full body to see what people are doing.

Similarly, physicians also missed important nonverbal information, such as lack of eye contact, repetitive movements, or hygiene issues, which may have impacted their ability to accurately diagnose and gauge patient comprehension. For those with hearing impairments, it was sometimes more difficult to understand the providers, especially if they had a heavy accent or if there was no visual component. Conversely, the chat function, if available, can offer a valuable alternative way to communicate for some individuals. Additionally, despite the limitations of video, it was sometimes a better option to see faces while mask requirements were in effect for in-person visits.

Despite the value of having multiple people participate in the visit, it sometimes made it more challenging to manage the conversation, especially by phone. One physician shared:

It's almost impossible on the phone to capture both voices. […] Very, very difficult to really get that triadic relationship and back and forth on the speakerphone just because everyone struggles to know when to talk and [we] have no visual cues.

Participants also reported that it can be more difficult to develop rapport over virtual interactions, particularly when the visit is with a new primary care provider. People with IDDs can have more difficulty engaging in virtual interactions and may be easily distracted when in their own homes. Without good communication and rapport, patients may be less interested in participating and less likely to disclose health issues, and important health issues may be missed.

##### Seeing Patients at Home

In-person visits can be an overwhelming and stressful experience for some people with IDDs, causing distress for the individual and leading to a less effective appointment. Allowing the patient to participate from their home where they are calm and comfortable can improve the quality of the visit. One physician explained:

For my patients who don't enjoy coming into clinic, it's very stressful for them in terms of the sensory stimuli [and] in terms of the social interaction. I found that being on video, or just by phone even, I'm able to get a lot more history from them and a lot more engagement in that discussion than if they were in office because they're so overwhelmed and so just preoccupied with being in the office that there really isn't that much bandwidth to engage.

Participants shared that in some cases, virtual care allowed them to reduce or eliminate use of medications that were previously needed to get the patient to the appointment. Video visits have the additional benefit of allowing primary care providers to see how patients act in their home environments, which can be important to inform appropriate treatment.

##### Importance of the Physical Examination

Participants stressed that in-person physical examinations will always be a necessary component of care. It was suggested that for some adults with IDDs, the physical examination is even more important because they are unable to describe their symptoms. One physician shared:

I think what ended up happening was I just, I really wasn't trusting my virtual assessments in the same way that I might in someone in the general population. So I was often bringing them into clinic, just feeling like I had to do the clinical exam to complete my assessment and honestly, just to reassure myself of my virtual assessment.

There are some types of primary care visits that must be conducted in-person but others that may be possible to conduct virtually depending on a range of factors, including the capacity of the caregiver to support an examination or administer treatment, the feasibility of a quality video visit, the patient’s level of insight into their physical health, and the patient’s ability to communicate virtually.

##### Privacy and Confidentiality

Patients who lived with the family or in supported settings did not always have a private space from which to participate in the primary care visit. Primary care providers expressed concern that they did not always know who else was in the room or able to listen to the visit. There were also concerns about cyber security for medical information shared online. It was noted that this patient population may be particularly vulnerable to scams and may end up disclosing medical information to predators.

##### Safety

Participants highlighted that an important benefit of virtual care was infection control. This was critical during COVID-19 but could also be an important ongoing benefit beyond the pandemic. This was also particularly relevant for some people with IDDs who could not tolerate wearing masks. There were some circumstances, however, when participants felt that in-person care was safer, such as when discussing topics that might trigger thoughts of self-harm. The father of a 22-year-old autistic man with complex mental health needs shared:

In person, there's the safety factor. If there's something that's been triggering for [my son], I think he feels safer if there's somebody else physically there.

#### Contextual Factors

Across the 4 access-to-care dimensions, we found that virtual care can be accessible for some individuals under certain circumstances. The success or appropriateness of a virtual care encounter is dependent on the interaction between 5 categories of factors. First, the characteristics of the individual patient, including their communication ability or style, income, skill and comfort using technology, difficulty attending in-person appointments, and visit frequency. Second, the patient’s context, including their distance from the health care service, the local internet quality, and access to a private space to conduct the appointment. Third, the characteristics of the caregiver, including their skill and comfort using technology, income, distance from the patient, and other responsibilities (eg, multiple caregiving roles). Fourth, the service context, including the usability of the virtual platform, the provider reimbursement model, the provider’s skill and comfort using technology, and whether the provider has a pre-existing relationship with the patient. Fifth, the specific reason for a particular health care visit, for example, if the visit requires a physical examination or a more complex or triggering discussion. Some of these variables are fixed, but many may change over time or per individual health care visit.

## Discussion

### Principal Findings

This qualitative study explored the accessibility of virtual primary care for patients with IDDs during the COVID-19 pandemic. A key finding was that one size does not fit all. We identified themes across 4 dimensions of accessibility that highlighted both the benefits and challenges of virtual care. For some patients, virtual delivery was critical to accessing necessary health care; for other patients, virtual delivery posed a barrier to accessing high-quality care. Whether virtual care was accessible was dependent on a combination of factors, including the characteristics of the patient, the patient’s context, the characteristics of the caregiver, the service context, and the reason for the specific health care visit. Some of these variables may be relatively constant (eg, patient’s communication style), some may change over time (eg, comfort with technology, distance from the provider, and private space), and some may change per appointment (eg, need for a physical examination).

Many of the themes identified in this study align with those identified in previous studies focused on the general patient population. Virtual care had many benefits, especially for simple issues or follow-up care. The benefits included greater convenience for patients, lower risk of COVID-19 transmission, and the value of seeing patients in their home environment [1,6,44,51]. Virtual care was not appropriate for issues that required a physical examination, and while some issues could be diagnosed via photographs or video observations, this could be challenging due to technology failures, lack of nonverbal communication, privacy concerns, and difficulties establishing rapport with new patients [1,6,44,51].

This study showed, however, that these same issues may look different or require different accommodations for patients with IDDs. One important difference for patients with IDDs is that there is often a caregiver involved, leading to additional considerations for virtual care, such as the caregiver’s comfort using technology, the value of facilitating the caregiver’s participation if they live far away, and difficulties managing calls with multiple participants. Communication and rapport in virtual care may be challenging for many individuals but can be additionally challenging for individuals with IDDs who are nonverbal, use communication devices, or have trouble engaging in virtual interactions. The physical examination is a critical component of care for all patients, but it can play a larger role in the care of people with IDDs if they are unable to identify or articulate their symptoms. Privacy may be a concern for anyone participating in a health care visit from their home, but it can be a particular concern for people with IDDs who live in congregate settings. Virtual care may be convenient for many patients, but for some people with IDDs, it is a necessary accommodation to support access to care.

The fifth access dimension, approachability, was not identified in this study but is nonetheless important to consider in designing accessible primary care systems. There has been a great deal of inconsistency in how primary care has been delivered during COVID-19, and patients and caregivers are likely unaware that it is possible to receive care in different ways than offered by their primary care provider. Direct education is needed for patients and caregivers to ensure they know what is available, what they are entitled to, and how to advocate for it.

It is important to note that some of the challenges and benefits of virtual care identified in this study are specific to the current context and may be less relevant moving forward. Some of the benefits of virtual care are related to COVID-19, including reducing the risk of COVID-19 transmission and accommodating patients who cannot tolerate wearing masks. Additionally, some of the challenges are because virtual care was implemented abruptly, with little existing infrastructure to deliver it effectively, for patients with little experience participating in virtual care. In this study, some participants noted that at the time of their interview, at least 1 year into the pandemic, they already felt more comfortable and proficient using technology and participating in virtual care than they did at the start of the pandemic. People will likely continue to become more comfortable with virtual care as technology infrastructure improves and everyone becomes more accustomed to virtual interactions.

### Implementing Accessible Virtual Care

It is clear from the study findings that the relevant question is not whether virtual care is accessible, but how it can be implemented in a way that will promote and not hinder health care accessibility. This study suggests that an optimal system should include multiple modalities depending on patient need and preference, including in-person, telephone, video, and written communication options. Achieving this type of flexible patient-centered system requires policies and supports considering both care delivery and use.

From an implementation lens, the largest hurdle seems to be supporting video-based care. The vast majority of virtual care in Ontario has been delivered by telephone, with only a relatively small proportion of virtual care delivered by video [24,25]. This study suggested that while telephone care can be very effective in some cases, it also has a number of limitations, including communication for individuals with hearing impairments or who are reliant on nonverbal communication, developing rapport between patients and providers, and managing conversations with multiple participants. These limitations can lead to health issues being missed and patients with IDDs being less engaged or excluded entirely from the visit. While telephone and in-person options may be sufficient in many cases, this study suggests that video can play an important role in supporting accessible care and should be part of the basket of services available.

To support greater implementation of video-based care, it is important that patients and their caregivers have access to high-speed internet and video-enabled devices. During the pandemic, some health care organizations provided tablets and smartphones to low-income patients [52]. With appropriate funding, this is a practice that could be expanded across primary care practices. High-speed internet continues to be a challenge in parts of the province, and public investment is needed to build capacity [53]. Currently, multiple virtual platforms are used in the health care system, many of which are not user friendly. Ideally, a common virtual platform should be implemented across the system that is easy for patients, caregivers, and primary care providers to use; has built-in accessibility features (eg, chat box and captioning); and can be integrated into electronic medical record systems. Given that primary care providers may themselves struggle to use technology and do not have the time or expertise to provide technical support to patients and families, primary care practices would benefit from including a dedicated staff role to provide technical support [54,55]. This could include meeting patients at the beginning of the appointment to orient them to the platform and troubleshooting technical issues before the provider joins the session or even offering in-person practice sessions to teach patients and family members how to use the virtual platform.

Beyond building capacity for video-based care, we also need health care funding structures that facilitate and incentivize delivery of both in-person and virtual care options according to patient need and preference. There are unintended consequences with any reimbursement model, but it has been recommended that the best strategy to avoid incentivizing a particular modality to the detriment of patients is shifting from a fee-for-service model to capitation or salary-based payment models [44].

Primary care providers also need training on how to deliver care virtually. Currently, most providers have received little if any training on when virtual care is appropriate, how to work with patients to determine the most appropriate type of care per visit, and how to effectively deliver care using virtual modalities [56]. There is starting to be more focus on the importance of this topic in medical education [57-59], and it is critical that these programs include considerations for different patient groups, including patients with IDDs. It is important to also highlight that primary care providers generally receive little specific training on how to care for patients with IDDs [60-62]. Any virtual care–specific training should be situated within general competencies to provide high-quality care to patients with IDDs.

Efforts to support the implementation of high-quality accessible virtual care should be informed by the large body of literature on implementation and health technology. Frameworks, such as the nonadoption, abandonment, scale-up, spread, and sustainability (NASSS) framework developed by Greenhalgh et al [63], can help outline the many considerations, including but not limited to those identified above, required for successful adoption of virtual care.

### Strengths and Limitations

This is one of the few studies we are aware of that looks at the accessibility of virtual primary care for adults with IDDs. A strength of the study is the inclusion of a diverse sample, including patients, family members, IDD support staff members, and physicians, to elicit perspectives from a range of different experiences. However, this study only collected limited demographic data on participants, and we cannot speak about the diversity of participants in terms of race or other important intersectional identities. This study was conducted in English, and it would be important for future work to look at the impact of virtual care for non-English speakers who may have different experiences. The study was limited to individuals who had participated in at least one virtual primary care visit. Therefore, individuals unable, unwilling, or lacking the opportunity to participate in virtual care were not included. Most participating physicians had practices that included a focus on patients with IDDs, and their perspectives may differ from the perspectives of physicians less experienced in caring for this patient population.

The study was also limited to one Canadian province, and people in jurisdictions with different pandemic restrictions or different health care delivery systems may have had different experiences. This study reflects a single interview conducted with each participant during the second year of the pandemic. Though some participants described how their perspectives changed, we did not assess this directly. This study included perspectives from all members of the health care triad (patients, caregivers, and providers) but not related to the same encounter. It would be important for future studies to compare perspectives from the same encounter to understand how they may be similar or different.

All interviews were conducted virtually due to pandemic restrictions. Based on the interviewer’s observations, good rapport was developed with participants, and they generally provided positive feedback on the interview experience. However, some interviews were disrupted due to technical challenges. In 6 cases, these challenges were severe enough to require the interview to be completed by phone. It is possible that these challenges or other issues not immediately apparent to the interviewer (eg, lack of rapport or participant discomfort) may have impacted the quality of the information gathered.

### Conclusions

The COVID-19 pandemic has provided a unique opportunity to learn about the accessibility of virtual primary care for adults with IDDs. This study found that virtual care can increase the accessibility of primary care for some individuals with IDDs under some circumstances and decrease accessibility for others. To meet the needs of all patients, a flexible patient-centered approach is needed that includes in-person, phone, and video options. This system must be supported by the necessary infrastructure, resources, and supports to ensure that the potential benefits of virtual care can be fully realized. This includes training for patients, caregivers, and primary care providers; universal access to the technology necessary to participate in virtual care; implementation of accessible virtual care platforms; and a primary care funding structure that can facilitate and incentivize delivery of both in-person and virtual care. While virtual care is not appropriate or desirable for all patients, there is a subset of patients with IDDs for whom virtual care is not just convenient but can enable access to necessary health care.

## Abbreviations

IDD: intellectual and developmental disability

## References

[1] Breton M, Deville-Stoetzel N, Gaboury I, Smithman M, Kaczorowski J, Lussier M, Haggerty J, Motulsky A, Nugus P, Layani G, Paré G, Evoy G, Arsenault M, Paquette J, Quinty J, Authier M, Mokraoui N, Luc M, Lavoie M (2021). Telehealth in Primary Healthcare: A Portrait of its Rapid Implementation during the COVID-19 Pandemic. Healthc Policy.

[2] Glazier RH, Green ME, Wu FC, Frymire E, Kopp A, Kiran T (2021). Shifts in office and virtual primary care during the early COVID-19 pandemic in Ontario, Canada. CMAJ.

[3] Alexander GC, Tajanlangit M, Heyward J, Mansour O, Qato DM, Stafford RS (2020). Use and Content of Primary Care Office-Based vs Telemedicine Care Visits During the COVID-19 Pandemic in the US. JAMA Netw Open.

[4] Garattini L, Badinella Martini M, Zanetti M (2021). More room for telemedicine after COVID-19: lessons for primary care?. Eur J Health Econ.

[5] Murphy M, Scott LJ, Salisbury C, Turner A, Scott A, Denholm R, Lewis R, Iyer G, Macleod J, Horwood J (2021). Implementation of remote consulting in UK primary care following the COVID-19 pandemic: a mixed-methods longitudinal study. Br J Gen Pract.

[6] Imlach F, McKinlay E, Middleton L, Kennedy J, Pledger M, Russell L, Churchward M, Cumming J, McBride-Henry K (2020). Telehealth consultations in general practice during a pandemic lockdown: survey and interviews on patient experiences and preferences. BMC Fam Pract.

[7] Shaw J, Jamieson T, Agarwal P, Griffin B, Wong I, Bhatia RS (2018). Virtual care policy recommendations for patient-centred primary care: findings of a consensus policy dialogue using a nominal group technique. J Telemed Telecare.

[8] Mold F, Cooke D, Ip A, Roy P, Denton S, Armes J (2021). COVID-19 and beyond: virtual consultations in primary care-reflecting on the evidence base for implementation and ensuring reach: commentary article. BMJ Health Care Inform.

[9] Crawford A, Serhal E (2020). Digital Health Equity and COVID-19: The Innovation Curve Cannot Reinforce the Social Gradient of Health. J Med Internet Res.

[10] Starfield B, Shi L, Macinko J (2005). Contribution of primary care to health systems and health. Milbank Q.

[11] Services and Supports to Promote the Social Inclusion of Persons with Developmental Disabilities Act, 2008, S.O. 2008, c. 14. Government of Ontario.

[12] Lunsky Y, Klein-Geltink J, Yates E (2013). Atlas on the Primary Care of Adults with Developmental Disabilities in Ontario. ICES.

[13] Chadwick D, Wesson C, Fullwood C (2013). Internet Access by People with Intellectual Disabilities: Inequalities and Opportunities. Future Internet.

[14] Frielink N, Oudshoorn CEM, Embregts PJCM (2020). eHealth in support for daily functioning of people with intellectual disability: Views of service users, relatives, and professionals on both its advantages and disadvantages and its facilitating and impeding factors. Journal of Intellectual & Developmental Disability.

[15] Reed ME, Huang J, Graetz I, Lee C, Muelly E, Kennedy C, Kim E (2020). Patient Characteristics Associated With Choosing a Telemedicine Visit vs Office Visit With the Same Primary Care Clinicians. JAMA Netw Open.

[16] Stamenova V, Agarwal P, Kelley L, Fujioka J, Nguyen M, Phung M, Wong I, Onabajo N, Bhatia RS, Bhattacharyya O (2020). Uptake and patient and provider communication modality preferences of virtual visits in primary care: a retrospective cohort study in Canada. BMJ Open.

[17] Ziviani J, Lennox N, Allison H, Lyons M, Del Mar C (2009). Meeting in the middle: improving communication in primary health care consultations with people with an intellectual disability. Journal of Intellectual & Developmental Disability.

[18] Doherty AJ, Atherton H, Boland P, Hastings R, Hives L, Hood K, James-Jenkinson L, Leavey R, Randell E, Reed J, Taggart L, Wilson N, Chauhan U (2020). Barriers and facilitators to primary health care for people with intellectual disabilities and/or autism: an integrative review. BJGP Open.

[19] Nathawad R, Hanks C (2017). Optimizing the Office Visit for Adolescents with Special Health Care Needs. Curr Probl Pediatr Adolesc Health Care.

[20] Perry J, Felce D, Kerr M, Bartley S, Tomlinson J, Felce J (2014). Contact with primary care: the experience of people with intellectual disabilities. J Appl Res Intellect Disabil.

[21] Williamson HJ, Contreras GM, Rodriguez ES, Smith JM, Perkins EA (2017). Health Care Access for Adults With Intellectual and Developmental Disabilities: A Scoping Review. OTJR (Thorofare N J).

[22] Jones MC, McLafferty E, Walley R, Toland J, Melson N (2008). Inclusion in primary care for people with intellectual disabilities: gaining the perspective of service user and supporting social care staff. J Intellect Disabil.

[23] Selick A, Bobbette N, Lunsky Y, Hamdani Y, Rayner J, Durbin J (2021). Virtual health care for adult patients with intellectual and developmental disabilities: A scoping review. Disabil Health J.

[24] Bhatia RS, Chu C, Pang A, Tadrous M, Stamenova V, Cram P (2021). Virtual care use before and during the COVID-19 pandemic: a repeated cross-sectional study. CMAJ Open.

[25] Fu R, Sutradhar R, Li Q, Eskander A (2022). Virtual and in-person visits by Ontario physicians in the COVID-19 era. J Telemed Telecare.

[26] Harris L, Gilmore D, Hanks C, Coury D, Moffatt-Bruce S, Garvin JH, Hand BN (2022). "It was surprisingly equivalent to the appointment I had in person": Advantages and disadvantages of synchronous telehealth for delivering primary care for autistic adults. Autism.

[27] Chadwick D, Ågren KA, Caton S, Chiner E, Danker J, Gómez‐Puerta M, Heitplatz V, Johansson S, Normand CL, Murphy E, Plichta P, Strnadová I, Wallén EF (2022). Digital inclusion and participation of people with intellectual disabilities during COVID-19: A rapid review and international bricolage. Policy Practice Intel Disabi.

[28] Shaw SC, Davis L, Doherty M (2022). Considering autistic patients in the era of telemedicine: the need for an adaptable, equitable, and compassionate approach. BJGP Open.

[29] Levesque J, Harris MF, Russell G (2013). Patient-centred access to health care: conceptualising access at the interface of health systems and populations. Int J Equity Health.

[30] Sandelowski M (2000). Whatever happened to qualitative description?. Res. Nurs. Health.

[31] Sandelowski M (2010). What's in a name? Qualitative description revisited. Res Nurs Health.

[32] Bradshaw C, Atkinson S, Doody O (2017). Employing a Qualitative Description Approach in Health Care Research. Glob Qual Nurs Res.

[33] Chafe R (2017). The Value of Qualitative Description in Health Services and Policy Research. Healthcare Policy.

[34] Sullivan-Bolyai S, Bova C, Harper D (2005). Developing and refining interventions in persons with health disparities: the use of qualitative description. Nurs Outlook.

[35] Padgett DK (2012). Qualitative and Mixed Methods in Public Health.

[36] Palinkas LA, Horwitz SM, Green CA, Wisdom JP, Duan N, Hoagwood K (2015). Purposeful Sampling for Qualitative Data Collection and Analysis in Mixed Method Implementation Research. Adm Policy Ment Health.

[37] Beail N, Williams K (2014). Using qualitative methods in research with people who have intellectual disabilities. J Appl Res Intellect Disabil.

[38] Coons KD, Watson SL (2013). Conducting Research with Individuals Who Have Intellectual Disabilities: Ethical and Practical Implications for Qualitative Research. J Dev Disabil.

[39] Lloyd V, Gatherer A, Kalsy S (2006). Conducting qualitative interview research with people with expressive language difficulties. Qual Health Res.

[40] Caldwell K (2013). Dyadic interviewing: a technique valuing interdependence in interviews with individuals with intellectual disabilities. Qualitative Research.

[41] DeJonckheere M, Vaughn LM (2019). Semistructured interviewing in primary care research: a balance of relationship and rigour. Fam Med Community Health.

[42] COVID-19 Temporary Fee Schedule Codes Implemented-Physicians can begin to submit claims for COVID-19 on May 1, 2020. Ministry of Health.

[43] Kiran T, Glazier R, INSPIRE Team Association between virtual primary care and emergency department use: Preliminary Results. Map Health.

[44] Falk W The State of Virtual Care in Canada as of Wave Three of the COVID-19 Pandemic: An Early Diagnostique and Policy Recommendations. Canada.

[45] Durbin A, Balogh R, Lin E, Palma L, Plumptre L, Lunsky Y (2022). Changes in community and hospital-based health care use during the COVID-19 pandemic for adults with and without intellectual and developmental disabilities. J Intellect Disabil Res.

[46] Braun V, Clarke V (2006). Using thematic analysis in psychology. Qualitative Research in Psychology.

[47] Clarke V, Braun V (2018). Using thematic analysis in counselling and psychotherapy research: A critical reflection. Couns. Psychother. Res.

[48] Braun V, Clarke V (2014). What can "thematic analysis" offer health and wellbeing researchers?. Int J Qual Stud Health Well-being.

[49] Nowell LS, Norris JM, White DE, Moules NJ (2017). Thematic Analysis. International Journal of Qualitative Methods.

[50] Korstjens I, Moser A (2018). Series: Practical guidance to qualitative research. Part 4: Trustworthiness and publishing. Eur J Gen Pract.

[51] Gomez T, Anaya YB, Shih KJ, Tarn DM (2021). A Qualitative Study of Primary Care Physicians' Experiences With Telemedicine During COVID-19. J Am Board Fam Med.

[52] Digital Equity Call to Action: Bridging the Digital Divide. The Alliance for Healthier Communities.

[53] Ontario Internet Access Map. Connected North.

[54] Wisniewski H, Torous J (2020). Digital navigators to implement smartphone and digital tools in care. Acta Psychiatr Scand.

[55] Wisniewski H, Gorrindo T, Rauseo-Ricupero N, Hilty D, Torous J (2020). The Role of Digital Navigators in Promoting Clinical Care and Technology Integration into Practice. Digit Biomark.

[56] Donnelly C, Ashcroft R, Bobbette N, Mills C, Mofina A, Tran T, Vader K, Williams A, Gill S, Miller J (2021). Interprofessional primary care during COVID-19: a survey of the provider perspective. BMC Fam Pract.

[57] Jumreornvong O, Yang E, Race J, Appel J (2020). Telemedicine and Medical Education in the Age of COVID-19. Acad Med.

[58] Garber K, Gustin T (2022). Telehealth Education: Impact on Provider Experience and Adoption. Nurse Educ.

[59] Ha E, Zwicky K, Yu G, Schechtman A (2020). Developing a Telemedicine Curriculum for a Family Medicine Residency. PRiMER.

[60] Bowen CN, Havercamp SM, Karpiak Bowen S, Nye G (2020). A call to action: Preparing a disability-competent health care workforce. Disabil Health J.

[61] Trollor JN, Ruffell B, Tracy J, Torr JJ, Durvasula S, Iacono T, Eagleson C, Lennox N (2016). Intellectual disability health content within medical curriculum: an audit of what our future doctors are taught. BMC Med Educ.

[62] Wilkinson J, Dreyfus D, Cerreto M, Bokhour B (2012). "Sometimes I feel overwhelmed": educational needs of family physicians caring for people with intellectual disability. Intellect Dev Disabil.

[63] Greenhalgh T, Wherton J, Papoutsi C, Lynch J, Hughes G, A'Court C, Hinder S, Fahy N, Procter R, Shaw S (2017). Beyond Adoption: A New Framework for Theorizing and Evaluating Nonadoption, Abandonment, and Challenges to the Scale-Up, Spread, and Sustainability of Health and Care Technologies. J Med Internet Res.

